# Experiences with food insecurity and risky sex among low-income people living with HIV/AIDS in a resource-rich setting

**DOI:** 10.7448/IAS.18.1.20293

**Published:** 2015-11-04

**Authors:** Henry J Whittle, Kartika Palar, Tessa Napoles, Lee Lemus Hufstedler, Irene Ching, Frederick M Hecht, Edward A Frongillo, Sheri D Weiser

**Affiliations:** 1 Global Health Sciences, University of California, San Francisco (UCSF), San Francisco, CA, USA; 2 Division of HIV/AIDS, ID and Global Medicine, Department of Medicine, University of California, San Francisco (UCSF), San Francisco, CA, USA; 3 Department of Health Promotion, Education, and Behavior, University of South Carolina, Columbia, SC, USA; 4 Center for AIDS Prevention Studies, University of California, San Francisco (UCSF), San Francisco, CA, USA

**Keywords:** food insecurity, sexual behaviour, HIV, men who have sex with men, resource-rich, San Francisco Bay Area, homeless, marginally housed

## Abstract

**Background:**

Forty-nine million individuals are food insecure in the United States, where food insecurity and HIV/AIDS are prevalent among the urban poor. Food insecurity is associated with risky sexual behaviours among people living with HIV/AIDS (PLHIV). No qualitative studies, however, have investigated the mechanisms underlying this relationship either in a resource-rich setting or among populations that include men who have sex with men (MSM).

**Methods:**

Semi-structured in-depth interviews were conducted with 34 low-income PLHIV receiving food assistance in the San Francisco Bay Area. The interviews explored experiences with food insecurity and perceived associations with sexual risk behaviours. Interviews were conducted in English, audio-recorded and transcribed verbatim. Transcripts were coded and analyzed according to content analysis methods using an inductive-deductive approach.

**Results:**

Food insecurity was reported to be a strong contributor to risky sexual practices among MSM and female participants. Individuals described engaging in transactional sex for food or money to buy food, often during times of destitution. Participants also explained how food insecurity could lead to condomless sex despite knowledge of and desire to use safe sexual practices, largely because the need to obtain food in the short term was prioritized over the desire to use barrier protection.

**Conclusions:**

Our data extend previous research by demonstrating that food insecurity contributes to transactional and unprotected sex among urban poor individuals in a resource-rich setting, including among MSM. These findings underscore the importance of public health and social intervention efforts focused on structural inequalities.

## Introduction

Food insecurity, defined as “the limited or uncertain availability of nutritionally adequate, safe foods, or the inability to acquire personally acceptable foods in socially acceptable ways” [1], affects 49 million individuals in the United States, concentrated among low-income households [2]. HIV/AIDS in the United States also disproportionately affects the urban poor [3–5]. Studies indicate that many low-income people living with HIV/AIDS (PLHIV) regularly experience food insecurity [6–8]. Among PLHIV, food insecurity is associated with worsened immunologic and virologic outcomes, higher morbidity and mortality and increased risk of both vertical and horizontal transmission, including sexual transmission [1,9].

Population-based studies in diverse regions have shown that food insecurity is associated with inconsistent condom use [10–15], sexual exchange [10], multiple sexual partnerships [13,14,16], a lack of control in sexual relationships [10], sexual relations with older or gang-affiliated partners [16], sexual violence [17], symptoms consistent with sexually transmitted infections [12,15] and substance use before sex [16]. Some of these links were demonstrated in resource-rich settings [11,13,16]. In San Francisco, food insecurity was longitudinally associated with unprotected sexual activity and having multiple sexual partners among 154 homeless and marginally housed PLHIV [13].

Data from qualitative studies in sub-Saharan Africa corroborate these findings [18,19]. No qualitative studies investigating the relationship between food insecurity and risky sex, however, have been conducted in high-income countries. An in-depth understanding of the lived experience and precise mechanisms behind the quantitative associations found in resource-rich settings is therefore lacking. Limited data also exist on the relationship between food insecurity and risky sex among men who have sex with men (MSM). Here we aimed to address these research gaps by investigating the perceived effects of food insecurity on risky sexual behaviours among a population of low-income PLHIV in San Francisco and Alameda County, California.

## Methods

### Research setting and collaboration

The San Francisco Bay Area has high rates of both HIV/AIDS and food insecurity. At the end of 2014, there were 15,979 PLHIV in San Francisco, making up 2% of cases nationwide. Of these, 74% acquired HIV through MSM transmission [20]. Furthermore, over 90,000 residents of San Francisco were thought to be food insecure in 2013, with at least 12% of the city's population benefiting from direct food provision by non-profit organizations [21]. Despite this, food insecurity does not feature prominently in San Francisco's current HIV strategy. Several non-profit free-meal programs, however, actively target PLHIV. Our study participants were low-income PLHIV receiving food assistance from one such organization, Project Open Hand (POH), which provides take-home meals and groceries free of charge to chronically ill individuals. Clients with a physician-certified diagnosis of HIV/AIDS are provided differential services depending on symptomatology and life situation: Those classified as “mildly ill” by POH may receive either a weekly bag of groceries or up to seven pre-prepared meals each week; “severely ill” clients may receive both. Starting in 2013, the University of California, San Francisco (UCSF) partnered with POH to understand the relationship between food insecurity, food assistance and health.

### Study population

The study population was a group of PLHIV who had been recruited into, but had not yet initiated, a new POH pilot program named *Food=Medicine*, which provided three meals per day plus snacks to participating clients. PLHIV were recruited into the program by POH from its larger client base, with the criteria that they be HIV positive, over 18 years of age, English- or Spanish-speaking and low-income, with a history of good adherence to POH services. POH also aimed to maximize the diversity of gender, race/ethnicity, illness severity (mildly ill vs. severely ill) and location (San Francisco vs. Alameda County) in the program population and sought to include 30 to 35 PLHIV recruited on a rolling basis between April and June 2014.

### Recruitment strategy

PLHIV enrolled in the Food=Medicine program who had given permission to be approached for inclusion in our study were asked to participate. The only study inclusion criterion was participation in the Food=Medicine program, and there were no exclusion criteria. We recruited on a rolling basis during data collection until we reached saturation of ideas.

### Data collection

Semi-structured in-depth interviews were conducted with 34 study participants between April and June 2014. Participants additionally completed the validated US Department of Agriculture Household Food Security Survey; at the time of interview, 24 of the 34 participants were food insecure, and 17 of the 34 were severely food insecure (i.e. “very low food security”). After collecting demographic information on gender, sex, race/ethnicity, education, housing and marital status, interviews were conducted in English and followed an interview guide exploring finances, life situation, food security, physical health, mental health, sexual health, engagement in HIV care and perceptions of POH. Questions related to sexual behaviour explored sexual relationships, sexual practices including use of barrier protection, difficulties declining sexual advances, experiences of transactional sex and concurrent sexual partners and factors that impacted decision-making around sex. Interviews were audio-recorded with permission from participants and transcribed verbatim. Participants were provided with $20 cash at the end of their interview.

### Data analysis

Transcripts were coded and analyzed using content analysis methods [22,23], following an integrative inductive-deductive approach [24]. A study team of five researchers developed a list of codes during data collection from both the interview guide and a preliminary review of the data as it was being gathered. This approach provided us with an organizing framework for the codes while also allowing new codes to emerge from the interview transcripts, which were read and discussed by the study team as interviews were conducted. The list of codes was then refined into a codebook. The primary interviewer coded all transcripts using the qualitative text management software Dedoose. Double-coding took place at predetermined intervals (every fourth transcript; *n*=8), with discrepancies discussed and resolved by consensus within the study team to validate the codebook and maximize coding reliability. The study team then discussed excerpts captured by the codes until consensus to identify salient themes. Selected quotations were chosen to illustrate key themes and sub-themes.

### Ethics statement

UCSF's Committee on Human Research granted the study ethical approval in January 2014. Participation was entirely voluntary and had no impact on either the receipt of standard POH services or Food=Medicine program participation. Informed written consent was obtained from all participants.

## Results

The sample consisted mostly of men (*n*=28 of 34), consistent with POH's HIV-positive client base. Twenty-six participants were living in an apartment or house at the time of interview, six were residing in single room occupancy hotels and two were staying with friends. Only five participants were married or living with a partner. Education level was generally high among participants: 30 had completed secondary education, and 25 had at least some college education (Table 1).

**Table 1:** Participant demographics

	*n*	%
Age		
39–45	4	12
46–55	16	47
56–65	11	32
66–70	3	9
Gender		
Male	28	82
Female	6	18
Disease severity		
Mildly ill	17	50
Severely ill	17	50
Residence		
San Francisco	21	62
Alameda County	13	38
Race/ethnicity*		
White/Caucasian	17	50
Black/African American	16	47
Asian	0	0
Hispanic	6	18
Other	5	15
Highest level of education completed		
Primary school	4	12
High school	3	9
General Educational Development (GED)	2	6
Some college	14	41
College – undergraduate	7	21
College – graduate	4	12
Current housing status		
Apartment or house	26	76
SRO or nightly hotel	6	18
Staying with friends	2	6
Marital status		
Married	2	6
Widowed	3	9
Divorced	9	26
Separated	1	3
Never married	15	44
Living with partner	3	9
Other	1	3

* Participants could self-identify as multiple categories. SRO, single room occupancy.

During the interviews, most participants described experiencing financial hardship and food insecurity, which included periods of insufficient quantity of food, long-term struggles with poor quality diets and having to procure food using personally and socially unacceptable strategies. About two-thirds of men described having sexual relationships with other men, while most women described having primarily heterosexual relationships. Two key themes emerged linking food insecurity with risky sex. The first was the use of transactional sex as a strategy for procuring food; the second was engagement in condomless sex because of food insecurity.

### Transactional sex as a procurement strategy

Both male and female participants described how they had engaged in sexual activity with men during times of severe food insecurity with the express purpose of procuring either food or money to buy food. Participants who had been forced to rely on such encounters described transactional sex in unfavourable terms. One female participant who had been homeless for some years had lived through periods of severe food shortage, during which she had sometimes turned to transactional sex with casual partners when unable to buy her own meals:

Yes, I have [engaged in transactional sex]. Basically, you know, we have all done things that we are not proud of, but yeah, for a meal you've got to do what you've got to do sometimes. It's not the nicest thing, it's a shame. But when your stomach's talking to you, you kind of give in to mercy.

All narratives of transactional sex motivated by food insecurity were associated with an absolute lack of food, that is, with salient experiences of hunger. Only the most severe form of food insecurity, in other words, led to sexual exchange among our participants, as illustrated by this female participant:

That [engaging in transactional sex] was before I even knew I had this [HIV], you know, and I was younger, maybe like in my twenties or something. Like out there trying to survive in the streets. By myself, being hard-headed. And I came from a good family. And I should've just went home. Instead of being stupid. Hanging with the wrong crowd, you know? Yeah. Oh, I've felt hungry before. I've been so hungry that my back, it felt like my stomach was touching my back. That's hunger.

Many participants had lived through times of homelessness in their lives, and they often described experiencing severe food insecurity and hunger during these periods. Accordingly, many narratives of transactional sex were associated with destitution. One male participant described a long stretch of homelessness in San Francisco during which street hustling – including transactional sex with other men for both food and money to buy food – had constituted his primary livelihood:

There was plenty of times that I did [sexual] things with guys just to know that I'd get something to eat and at least a couple of bags [of marijuana], that I wouldn't have probably normally done, except I needed something to eat and I didn't have any money at all, and I didn't have any resources other than that to turn to …. Oh yeah, there was a number of times that that was why we engaged, so at least I knew I'd eat, anyway. And have some place to sleep.

This participant also revealed that transactional sex with other men, as opposed to with women, was the only viable form of sex work he could perform during his time on the streets:

[Transactional sex was] with other men, yeah. God, I wish I could've done it with women. I never found that racket. I looked for it a couple of places, I heard it happened, but no.

Reflecting on a three-year period of homelessness and severe food insecurity that occurred while waiting for public housing after losing his job due to illness, one male participant described his motivations for employing undesirable survival strategies – including exchanging sex for food with other men – as follows:

I mean there are things that I understand… People are really interesting, and I understand more so what makes a person do something. Sometimes, not that they always agree with it, but sometimes they're left no choice. There are some things still that I – I would never steal from someone, never rob anyone, never kill anyone, you know, so I don't understand those things still. But I understand how to meet basic needs sometimes, how people are put in situations or put themselves in situations that they have to do something that they don't really want to. But in order to survive they have to, and I see it all the time. I've experienced it.

A key dimension of participants’ narratives on transactional sex concerned the process by which sexual encounters arose. Two sub-themes emerged here: transactional sex as a livelihood strategy and transactional sex as an opportunistic encounter. In the first instance, some participants described engaging in sexual exchanges as a more considered – and sometimes long-term – strategy that could reliably provide them with food. This narrative is epitomized by individuals who perceived themselves as engaging in sex work, describing their exchanges as “prostitution” or “hustling.” Also falling under this sub-theme were the experiences of a male participant who had recently experienced a long period of severe food insecurity because his monthly state income for work-limiting disability was only just enough to cover his rent payments. While not perceiving his exchanges as sex work, this participant described actively searching for male sexual partners with the explicit purpose of obtaining food:

I've played on and dated people just to go out with them to eat and get money, you know? I've done it just last year. Yeah, I've done it, yeah. And I probably would do it again if push comes to shove; I'm not going to say I won't. But I try to avoid that situation. I really do.

Other individuals described their sexual exchanges as more opportunistic encounters. These occurred primarily during times of homelessness, when participants had been on the streets and opportunities had arisen for sexual encounters with strangers that might provide them with food. Obtaining shelter was often evoked as another primary motivation in this type of situation. A male participant described this approach and differentiated his sexual exchanges from sex work:

It wasn't that I was like actually hustling or advertising for it. It was just more kind of like desperate times, desperate measures, you know?

The line between these two sub-themes, however, could be blurry; some participants described habitually using opportunistic encounters as a means of alleviating the more salient negative aspects of homelessness. This was the situation for a male participant during a long period of homelessness and severe food insecurity:

Whatever you're getting out of it, or whatever they [the sexual partner] are getting out of it, it may be two different reasons that you need whatever. But you each have your own reason. Again, it goes back to doing something that maybe you normally wouldn't do, but because you're tired or you're sleepy or you're hungry or you're whatever, yeah, you do it.

Finally, some participants explained that their experiences with transactional sex for food had sometimes compromised their safety. For individuals who had used sex work as a livelihood strategy, this included engaging with clients with whom they did not feel comfortable. A female participant, who explained that a lack of food had motivated her decision to engage in sex work, described this kind of situation:

[I did things I felt uncomfortable with for money] a few times. Not often, but a few times. Yeah, I was a prostitute for a long time, but usually if I didn't feel comfortable with it I would just walk out. I would say, “Fuck the money,” pardon my French. But a few times.

Similarly, a male participant described how sexual encounters with male strangers, motivated by a desire for food and shelter while he was homeless, had sometimes put him in physical danger:

You hope that you are cautious enough about certain things, but sometimes you just can't even think about that. Sometimes you're just like, “I just want to eat and I just want to sleep,” you know? But there are [sexual] things you have to do beforehand or whatever …. And all that stuff is more important than thinking about if something bad is going to happen or if he's going to, you know, kill me in my sleep or rob me. Because people have stolen from me, I've been robbed, yeah, you name it. But at the time it doesn't matter.

### Food insecurity leading to condomless sex

Participants across the sample were well informed about safe sexual practices and most expressed that they preferred to use condoms during sexual encounters, particularly with non-primary partners. Moreover, participants reported that they were generally able to successfully insist on using barrier protection whenever they wanted to during sexual encounters that were not motivated by a lack of resources. During times of food insecurity, however, the situation was more variable. Some individuals who had engaged in transactional sex for food reported that they were always able to insist on using barrier protection during such encounters. This was the case for a female participant whose primary motivation for protected sex was preventing pregnancy:

No, I have always used [barrier] protection [during transactional sex]. Yeah, that's what they are going to do. Because I am not going to have no more babies out of wedlock, you know what I'm saying? That's just the thing. I'm the one stuck with the baby. So that's the least they can do, is use protection.

Other participants, however, described how food insecurity could sometimes compromise their ability to insist on using barrier protection. Participants only reported that this had occurred during transactional sexual encounters when they were experiencing the most severe form of food insecurity, that is, food shortages with resultant hunger. Specifically, the need to obtain food in the short term was prioritized over the desire to use barrier protection. Sometimes this was at the insistence of the sexual partner, as described by a female participant who had engaged in condomless sex during sex work and been influenced by hunger in making this decision:

There were a few times [when I didn't use barrier protection when I otherwise would have], which is probably how I got infected. Where I got offered more money not to use a condom, and to be quite frank with you, I did take the money.

Other participants explained that the effects of hunger could skew normal processes of prioritization, pushing the use of barrier protection out of their minds during sexual exchanges. A male participant who had habitually used opportunistic sexual encounters with other men to obtain food and shelter during a period of homelessness described this mechanism:

You're not thinking about [using barrier protection], or you're trying not to think about it, like I said, because the other needs are higher up the priority list than that one. How can that be? I don't know, but yeah, it is. Your body and mind does other things when it's hungry, when it's tired. Because you're not thinking right. You're not getting the sleep and the food that you need to function. Nothing works right. So it's like you just have to pick and choose then.

## Discussion

MSM and female participants described engaging in transactional sex with casual partners in response to severe food insecurity as a means of procuring food or money to buy food, most often during periods of destitution. Both sex work and unplanned opportunistic encounters were described, and individuals explained how their safety had sometimes been compromised during these engagements. Moreover, participants articulated that food insecurity could lead to condomless sex with casual partners during transactional encounters, when the need for food in the short term was prioritized over the desire to use barrier protection.

Qualitative studies have previously described mechanisms for how food insecurity contributes to HIV acquisition risk in resource-poor settings. HIV-positive Ugandan women explained how the experience or threat of hunger could lead to transactional sex for food with men [18]. Sex workers in Swaziland described food insecurity as a primary motivation for both joining and remaining in the trade [19]. In these studies, the provisional role of male sexual partners allowed them to insist on unprotected sex against the wishes of the women [18,19]. Our data extend this research by (1) corroborating these mechanisms of food insecurity driving transactional and condomless sex in a resource-rich setting and (2) documenting similar mechanisms for the first time among MSM.

A notable contrast to findings in resource-poor contexts [18] is that long-term transactional relationships were not a major theme in our results. Several of our participants were or had previously been in long-term cohabiting relationships, but almost none described transactional sexual dependence in such arrangements – usually because pooling two low incomes (e.g. disability benefits) was sufficient to avoid the levels of destitution and severity of food insecurity driving transactional sex in this setting. This pattern of results is consistent with the finding that 91% of homeless individuals in San Francisco in 2015 were single adults without families [25], as well as evidence that suggests destitution and food insecurity can result from an imbalance between single low incomes and urban rent prices [26,27].

Narratives from sub-Saharan Africa were also strongly influenced by gender inequality: women described a patriarchal culture in which it was often difficult to insist on condom use against the wishes of the male partner, regardless of (but exacerbated by) resource struggles [18]. In contrast, participants in our study explained that they were generally able to use barrier protection whenever they wanted to, except during times of severe food insecurity and destitution. The power imbalance at play during risky sex, therefore, was primarily one of socio-economic inequality in our findings, characterized by financial non-viability in a region of relative wealth. Nonetheless, it is notable that all experiences of transactional sex here, whether described by male or female participants, involved exclusively male sexual partners, despite the presence of several men in the sample who expressed a sexual preference for women. This highlights the particular vulnerability of MSM and women to risky sex motivated by food insecurity, either as sex work or opportunistic encounters co-driven by homelessness.

Our study has limitations. Firstly, the POH recruitment criteria into the Food=Medicine program required that enrolees have a history of good compliance with prior services, which may have filtered out clients with particularly unstable life situations such as issues of substance dependency or chronic homelessness. Secondly, given that recruitment proceeded among individuals who were receiving food assistance, we may have missed PLHIV who use transactional sex or relationships as a more prominent, long-term source of food. Given these limitations, however, it is notable that themes of risky sex arising from food insecurity were still highly salient even in this relatively more stable population.

## Conclusions

Our findings emphasize the need to address food insecurity as a part of public health and social intervention efforts against the HIV/AIDS epidemic in high-income countries, especially in regions of relatively concentrated urban poverty and destitution such as the San Francisco Bay Area. Knowledge of safe sexual practices and desire to engage in protected sex were common in our study, and yet the agency of participants was constrained by larger economic forces that led to transactional and condomless sex to obtain food. This indicates a need for interventions addressing food insecurity and other forms of financial deprivation to complement purely educational programs against risky sex. Comprehensive food assistance interventions such as the Food=Medicine program, coupled with quantitative process and impact evaluation using experimental or quasi-experimental designs, are an appropriate starting point to provide evidence in support of structural change.

## References

[1] Anema A, Vogenthaler N, Frongillo EA, Kadiyala S, Weiser SD (2009). Food insecurity and HIV/AIDS: current knowledge, gaps, and research priorities. Curr HIV/AIDS Rep.

[2] Coleman-Jensen A, Gregory C, Singh A (2014). Household food security in the United States in 2013.

[3] Robertson MJ, Clark RA, Charlebois ED, Tulsky J, Long HL, Bangsberg DR (2004). HIV seroprevalence among homeless and marginally housed adults in San Francisco. Am J Public Health.

[4] Beijer U, Wolf A, Fazel S (2012). Prevalence of tuberculosis, hepatitis C virus, and HIV in homeless people: a systematic review and meta-analysis. Lancet Infect Dis.

[5] Pellowski JA, Kalichman SC, Matthews KA, Adler N (2013). A pandemic of the poor: social disadvantage and the U.S. HIV epidemic. Am Psychol.

[6] Weiser SD, Bangsberg DR, Kegeles S, Ragland K, Kushel MB, Frongillo EA (2009). Food insecurity among homeless and marginally housed individuals living with HIV/AIDS in San Francisco. AIDS Behav.

[7] Vogenthaler NS, Hadley C, Lewis SJ, Rodriguez AE, Metsch LR, del Rio C (2010). Food insufficiency among HIV-infected crack-cocaine users in Atlanta and Miami. Public Health Nutr.

[8] Anema A, Weiser SD, Fernandes KA, Ding E, Brandson EK, Palmer A (2011). High prevalence of food insecurity among HIV-infected individuals receiving HAART in a resource-rich setting. AIDS Care.

[9] Weiser SD, Young SL, Cohen CR, Kushel MB, Tsai AC, Tien PC (2011). Conceptual framework for understanding the bidirectional links between food insecurity and HIV/AIDS. Am J Clin Nutr.

[10] Weiser SD, Leiter K, Bangsberg DR, Butler LM, Percy-de Korte F, Hlanze Z (2007). Food insufficiency is associated with high-risk sexual behavior among women in Botswana and Swaziland. PLoS Med.

[11] Shannon K, Kerr T, Milloy MJ, Anema A, Zhang R, Montaner JS (2011). Severe food insecurity is associated with elevated unprotected sex among HIV-seropositive injection drug users independent of HAART use. AIDS.

[12] Tsai AC, Hung KJ, Weiser SD (2012). Is food insecurity associated with HIV risk? Cross-sectional evidence from sexually active women in Brazil. PLoS Med.

[13] Vogenthaler NS, Kushel MB, Hadley C, Frongillo EA, Riley ED, Bangsberg DR (2013). Food insecurity and risky sexual behaviors among homeless and marginally housed HIV-infected individuals in San Francisco. AIDS Behav.

[14] Eaton LA, Cain DN, Pitpitan EV, Carey KB, Carey MP, Mehlomakulu V (2014). Exploring the relationships among food insecurity, alcohol use, and sexual risk taking among men and women living in South African townships. J Prim Prev.

[15] Tsai AC, Weiser SD (2014). Population-based study of food insecurity and HIV transmission risk behaviors and symptoms of sexually transmitted infections among linked couples in Nepal. AIDS Behav.

[16] Raiford JL, Herbst JH, Carry M, Browne FA, Doherty I, Wechsberg WM (2014). Low prospects and high risk: structural determinants of health associated with sexual risk among young African American women residing in resource-poor communities in the south. Am J Community Psychol.

[17] Tsai AC, Leiter K, Heisler M, Iacopino V, Wolfe W, Shannon K (2011). Prevalence and correlates of forced sex perpetration and victimization in Botswana and Swaziland. Am J Public Health.

[18] Miller CL, Bangsberg DR, Tuller DM, Senkungu J, Kawuma A, Frongillo EA (2011). Food insecurity and sexual risk in an HIV endemic community in Uganda. AIDS Behav.

[19] Fielding-Miller R, Mnisi Z, Adams D, Baral S, Kennedy C (2014). “There is hunger in my community”: a qualitative study of food security as a cyclical force in sex work in Swaziland. BMC Public Health.

[20] San Francisco Department of Public Health (2015). HIV epidemiology annual report 2014.

[21] San Francisco Food Security Task Force (2013). Assessment of food security in San Francisco.

[22] Weber R (1990). Basic content analysis.

[23] Krippendorff K (2004). Content analysis: an introduction to its methodology.

[24] Bradley EH, Curry LA, Devers KJ (2007). Qualitative data analysis for health services research: developing taxonomy, themes, and theory. Health Serv Res.

[25] Applied Survey Research (2015). 2015 San Francisco point-in-time homeless count & survey.

[26] Dickson-Gómez J, Convey M, Hilario H, Weeks MR, Corbett AM (2009). Hustling and housing: drug users’ strategies to obtain shelter and income in Hartford, Connecticut. Human Organ.

[27] Whittle HJ, Palar K, Hufstedler LL, Seligman HK, Frongillo EA, Weiser SD (2015). Food insecurity, chronic illness, and gentrification in the San Francisco Bay Area: an example of structural violence in United States public policy. Soc Sci Med.

